# A Rare Case of Unilateral Impacted Permanent Maxillary Canine Tooth in an Inverted Position

**DOI:** 10.7759/cureus.23376

**Published:** 2022-03-21

**Authors:** Dyna Albert, Muthusekhar M.R., Santhosh P Kumar, Senthilmurugan M

**Affiliations:** 1 Oral and Maxillofacial Surgery, Saveetha Dental College and Hospital, Chennai, IND

**Keywords:** ectopic eruption, root resorption, retained deciduous teeth, dentigerous cyst, impacted canine

## Abstract

The probability of occurrence of impacted permanent maxillary canine tooth is 2% and, in most cases, is an incidental finding. The management options for them vary from no treatment, surgical exposure, and orthodontic traction into dental arch, surgical removal, and autotransplantation. Although orthodontic traction into the dental arch is the ideal treatment of choice, it cannot be achieved in permanent maxillary canines impacted in unfavorable or aberrant positions. In this article, we report a rare case of a unilateral impacted permanent maxillary canine tooth in an inverted position, which was an incidental finding during the patient’s routine dental examination and discuss the management options for impacted permanent maxillary canine teeth.

## Introduction

Permanent maxillary canines (PMCs) are the second most commonly impacted teeth, next to third molars. The incidence of impaction of PMC is 2% and is twice more common in females than in males [1]. Permanent canine teeth impaction manifests two times more in the maxillary jaw than in the mandible. Among the patients with impacted PMC teeth, only 8% are observed bilaterally [2]. One-third of the impacted maxillary canines are labially placed, while two-thirds are located in a palatal position [3]. A variety of factors can contribute to canine impaction, and the causes for palatally displaced maxillary canines are unknown [4]. It was found that 85% of the maxillary canines impacted palatally had enough space for eruption, whereas only 17% of the maxillary canines impacted labially had sufficient space for eruption. As a result, the disparity in the length of the dental arch is found to be the most common etiology in impacted maxillary canines placed labially [5]. Impaction of maxillary canines in an inverted position has an occurrence of only 0.6% among maxillary canine impactions and unilateral inverted maxillary canine impaction is rarer [6]. Localized, systemic, and genetic factors have been implicated for the occurrence of impacted PMC. This article aimed to report a rare case of a unilateral impacted PMC tooth in an inverted position and present/discuss the management options for impacted PMC teeth.

## Case presentation

**Figure 1:**
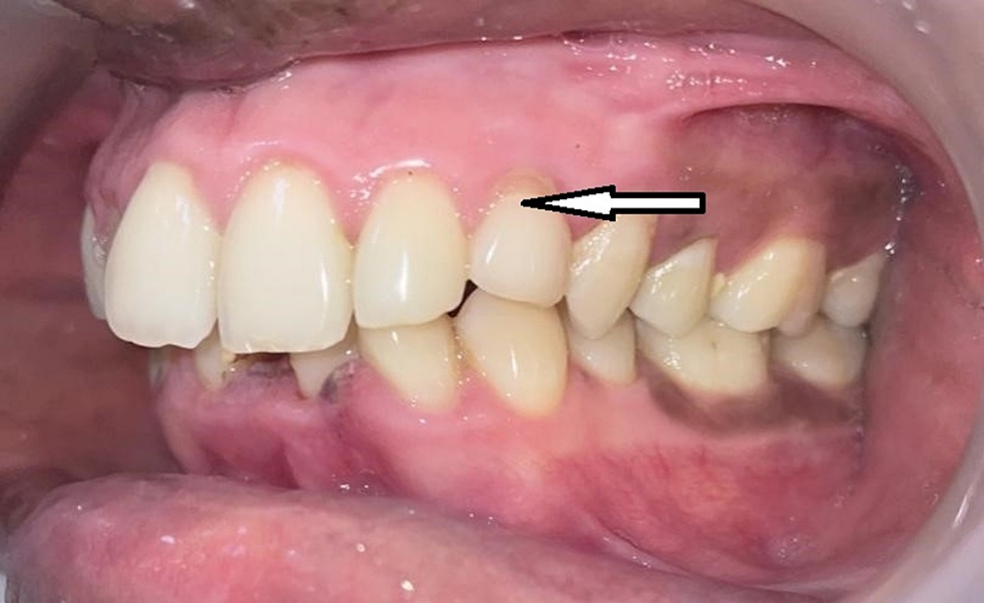
Intra-oral image showing retained left deciduous maxillary canine and missing left PMC PMC, permanent maxillary canine.

**Figure 2:**
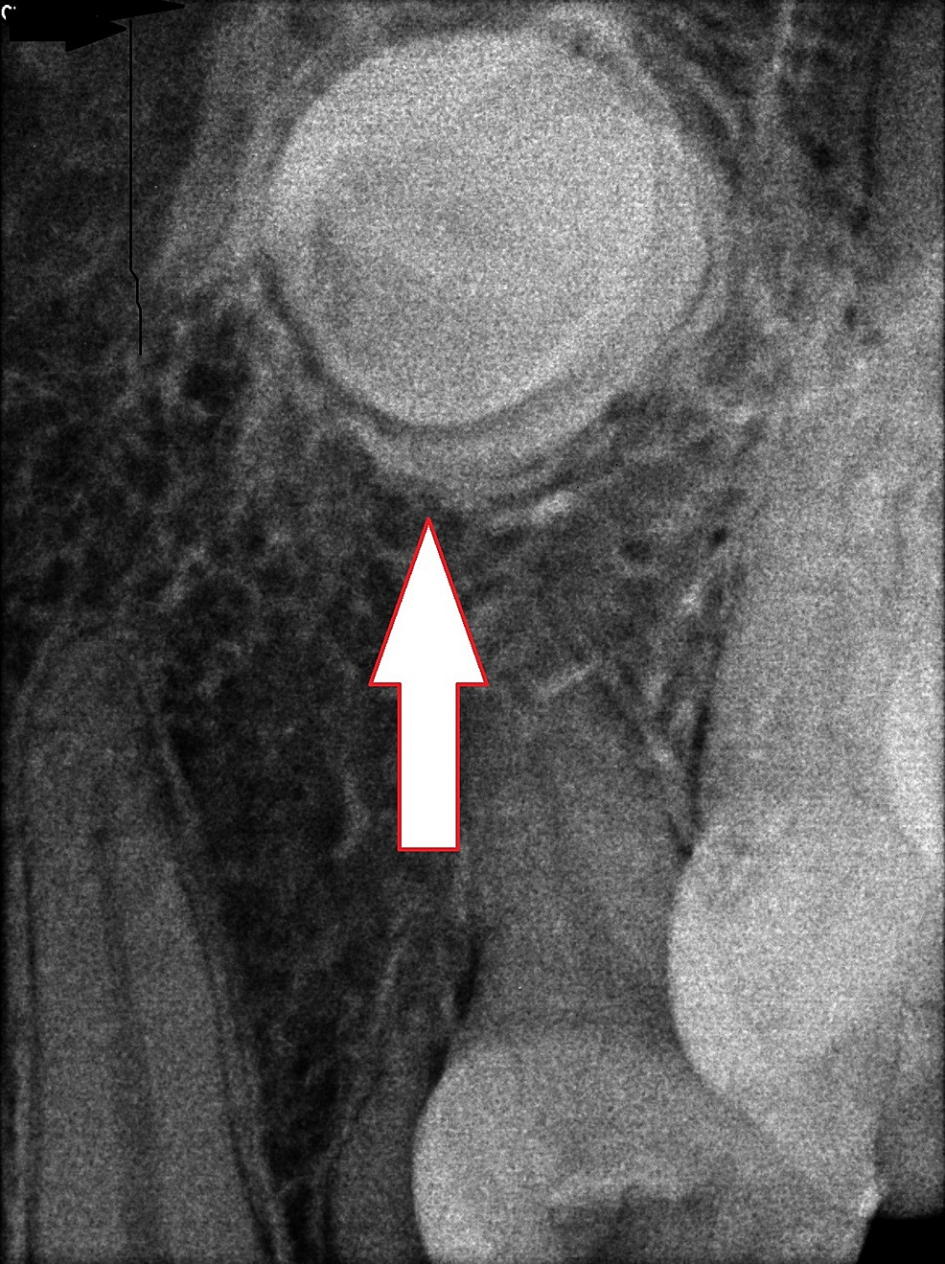
IOPA radiograph showing the presence of the impacted left PMC tooth IOPA, intra-oral periapical; PMC, permanent maxillary canine.

**Figure 3:**
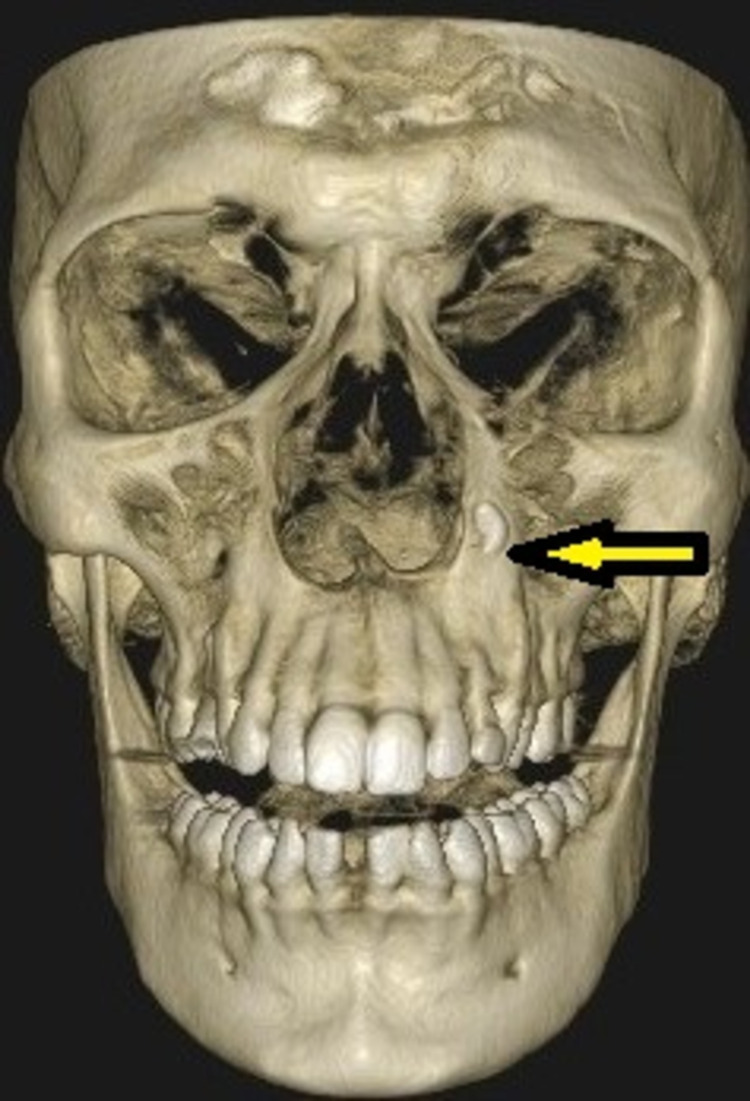
CBCT of facial skeleton showing the impacted left PMC tooth in an inverted position CBCT, Cone Beam Computed Tomography; PMC, permanent maxillary canine.

A 20-year-old male patient had reported to our institution for a general dental examination. Clinical examination revealed a missing left upper permanent canine tooth (23), a right lower third molar tooth (48), and a retained left upper deciduous canine tooth (63) (Figure 1). The patient was referred to the Department of Oral and Maxillofacial Surgery for further treatment. Radiological investigation with intra-oral periapical (IOPA) radiograph revealed the presence of impacted 23 (Figure 2). Cone Beam Computed Tomography (CBCT) revealed that the impacted 23 was inverted in position, located on the lateral of the nasal cavity and abutting the maxillary sinus on the left side (Figure 3). According to the orthodontist, orthodontic movement of the canine to the desired position was not feasible and surgical removal of the impacted 23 under general anesthesia was planned.

**Figure 4:**
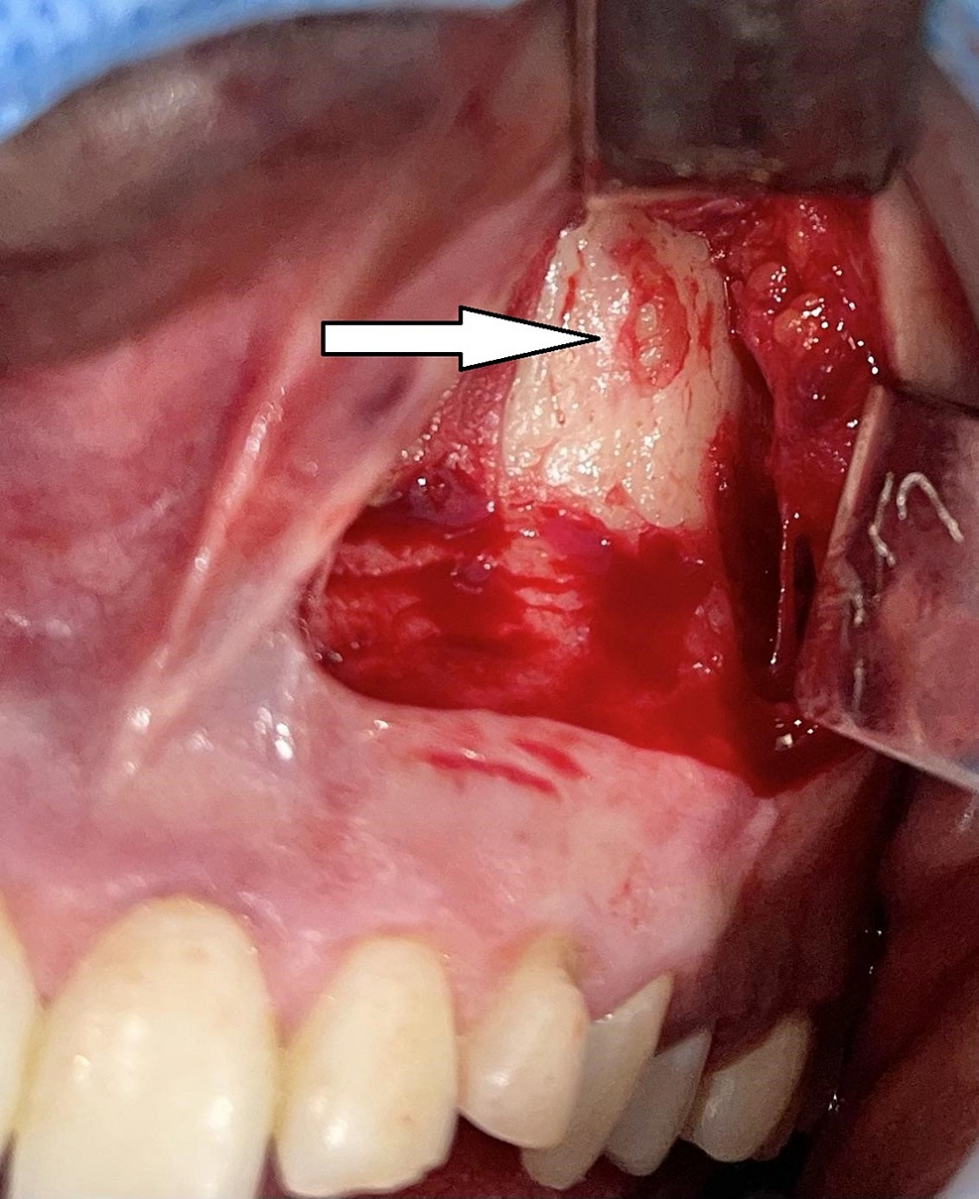
Surgical exposure revealing the bone covering the impacted left PMC tooth PMC, permanent maxillary canine.

**Figure 5:**
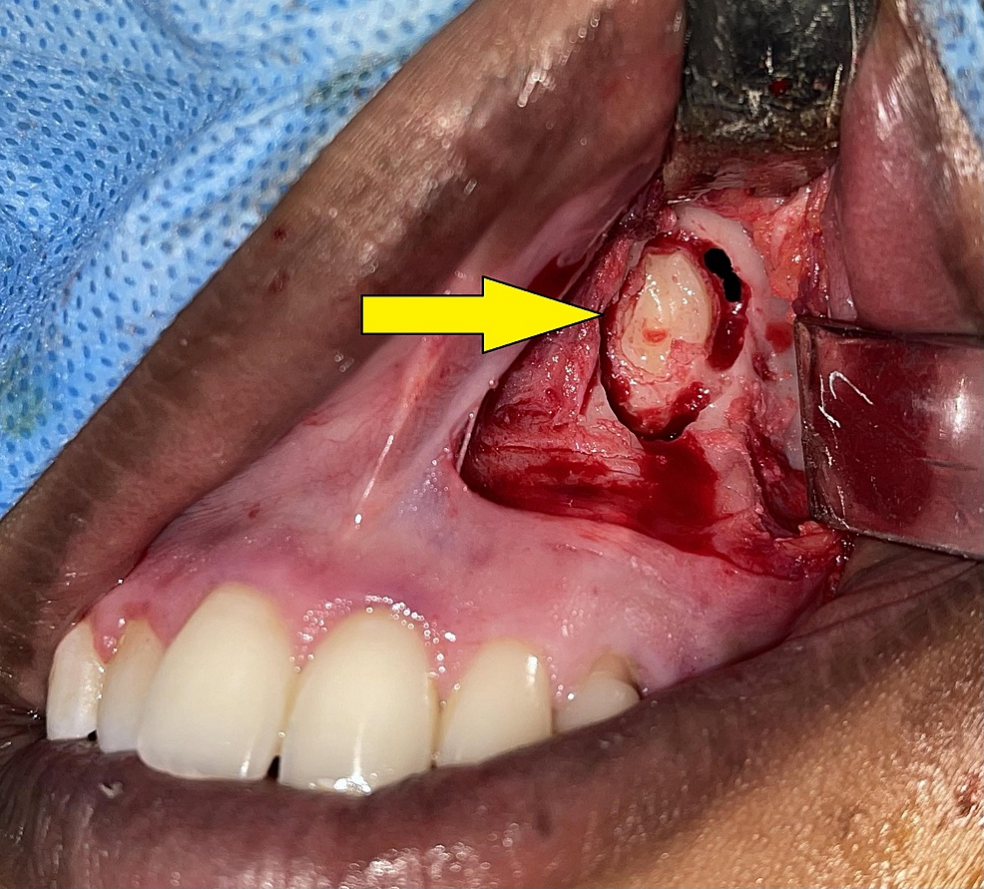
Surgical exposure of the impacted left PMC tooth with the crown facing upward and root downward PMC, permanent maxillary canine.

**Figure 6:**
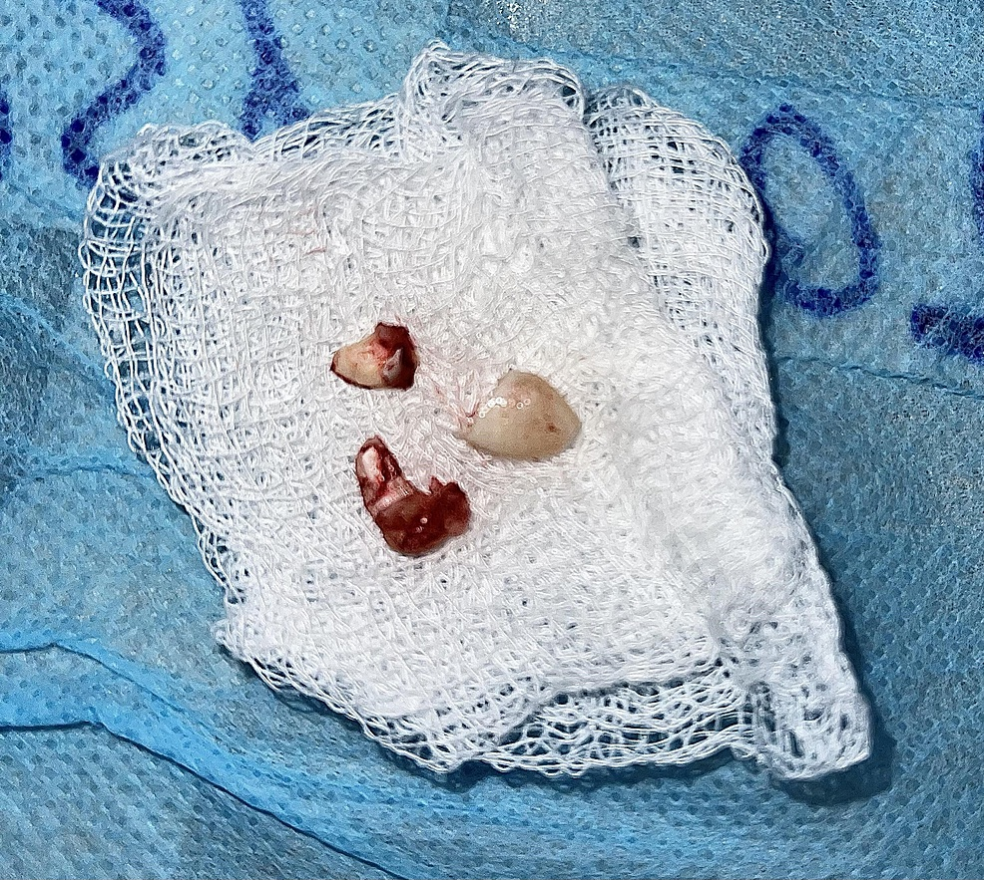
Extracted tooth portions

**Figure 7:**
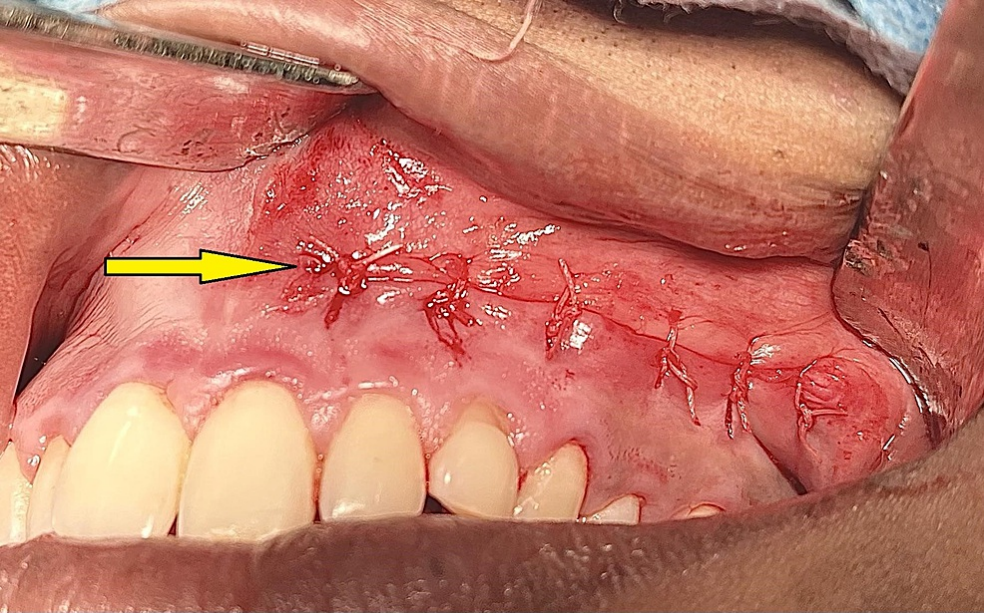
Closure of the surgical wound

Under general anesthesia, a vestibular incision was placed in the 21-25 region, and the mucoperiosteal flap was raised and bone got exposed (Figure 4). Bone guttering was done to expose the impacted maxillary permanent canine tooth (23), which was present in an inverted position with the crown facing upward and root downward (Figure 5). Tooth sectioning was done followed by the removal of crown and root separately (Figure 6). Antral packing was done in relation to the left maxillary sinus, hemostasis was achieved, and wound closure was done (Figure 7). Wound healing was uneventful in the postoperative period.

## Discussion

Localized, systemic, and genetic factors are major etiological factors for impacted PMC. Local factors that may contribute to PMC impaction include [7]:

1. Longer eruption path that the tooth must travel from its point of development to normal occlusion.

2. Thick palatal bone and mucoperiosteum can prevent the maxillary canines placed palatally from erupting.

3. Overdeveloped tooth roots during the eruption may reduce the forces of eruption.

4. Placement of the developing permanent canine’s crown palatal to the deciduous canine’s root apex.

5. Influence by environmental factors, as canines are the tooth that erupts last into position, except third molars.

6. Limited eruption space for maxillary canines, which may be further reduced by the second molar.

7. Narrower mesiodistal width of the permanent canine than that of the primary canine.

Many treatment options are available for impacted PMC. Factors that help in determining the treatment options include the patient’s age and medical status, the existence of accompanying pathology, stage of root development, degree of impaction, tooth position, the status of the dental arch and adjacent teeth, patient's cooperation to undergo orthodontic treatment, and the accessibility to treatment facilities [8].

If the patient is asymptomatic, regular clinical follow-up with radiographic assessments may be sufficient and no treatment may be required. However, it should be remembered that there is always a risk of the development of follicular cysts and odontogenic tumors [9]. Extraction of primary maxillary canine is a preventive management technique when the PMC is not visible or palpable in its desired position. The presence of impacted PMC must be confirmed radiographically before the extraction of the primary maxillary canine. However, further surgical treatment will be required if the PMCs do not erupt into their desired position within one year of extraction of primary maxillary canine [10,11].

Surgical exposure of impacted tooth is performed when the maxillary canine root growth is inadequate but sufficient dental arch space is available for it to erupt in the desired position. When the crown of the impacted PMC is surgically exposed, it allows normal eruptive forces to bring the tooth into the desired position [12]. Surgical exposure and orthodontically assisted eruption are better treatment options for impacted PMC and should be considered whenever feasible. Ideal requisites for this technique include the favorable position of an impacted canine, favorable medical condition of the patient, and adherence to both surgery and long-term orthodontics by the patient [13].

Surgical removal of the impacted teeth is the last treatment option. It is performed when the impacted maxillary tooth is in an unfavorable position and is likely to cause complications in the future [14]. It is also not uncommon to surgically remove the impacted PMC, regardless of its position if the patient is unwilling for other treatment options. In our case, as the impacted PMC was present in an inverted unfavorable position, it was surgically removed. An impacted PMC in an unfavorable position cannot be orthodontically aligned into occlusion and should be extracted surgically as early as possible at a younger age. As age advances, removal of the impacted teeth becomes difficult and there is a risk of development of pathology associated with impacted teeth [14]. Surgical repositioning or autotransplantation can be considered for impacted PMC that have good root patterns. Surgical repositioning of the impacted PMC can be done by autotransplantation of the canine into the extraction socket space of the first premolar or deciduous canine [15,16].

## Conclusions

Impacted PMC teeth rarely manifest in an inverted position. The presence of impacted PMC in an unfavorable inverted or aberrant position is extremely difficult to manage by surgical tooth exposure, orthodontic traction, and alignment into the dental arch. In such cases, surgical removal of the impacted PMC is the best treatment option.
